# Epigenetics in the pathogenesis of rheumatoid arthritis

**DOI:** 10.1186/1741-7015-12-35

**Published:** 2014-02-26

**Authors:** Tibor T Glant, Katalin Mikecz, Tibor A Rauch

**Affiliations:** 1 Section of Molecular Medicine, Department of Orthopedic Surgery, Rush University Medical Center, 1735 West Harrison Street, Chicago, IL 60612, USA

**Keywords:** Chromatin modifications, DNA methylation, Epigenetics, Rheumatoid arthritis

## Abstract

An increasing number of studies show that besides the inherited genetic architecture (that is, genomic DNA), various environmental factors significantly contribute to the etiology of rheumatoid arthritis. Epigenetic factors react to external stimuli and form bridges between the environment and the genetic information-harboring DNA. Epigenetic mechanisms are implicated in the final interpretation of the encoded genetic information by regulating gene expression, and alterations in their profile influence the activity of the immune system. Overall, epigenetic mechanisms further increase the well-known complexity of rheumatoid arthritis by providing additional subtle contributions to rheumatoid arthritis susceptibility. Although there are controversies regarding the involvement of epigenetic and genetic factors in rheumatoid arthritis etiology, it is becoming obvious that the two systems (genetic and epigenetic) interact with each other and are ultimately responsible for rheumatoid arthritis development. Here, epigenetic factors and mechanisms involved in rheumatoid arthritis are reviewed and new, potential therapeutic targets are discussed.

## Background

More than 10 years after the completion of the human genome sequencing project [1] and numerous genome-wide association studies (GWAS) [2], we still do not fully understand the genetic basis of rheumatoid arthritis (RA). GWAS on patients with RA revealed more than 30 genomic risk loci, but identification of disease-promoting genes and their functional characterization remain to be accomplished [3,4]. The delayed progress in RA genetics can be explained by the polygenic nature of the disease, the enormous genetic heterogeneity of the human population, and the difficulties with the interpretation of GWAS data since most of the significant genetic alterations (that is, mutations) are located in non-protein coding regions of the genome. Another observation that raises some doubt about a major role of genetic factors in RA pathogenesis is that the concordance rate in monozygotic twins is only approximately 15% [5]. However, twin studies drew attention to the importance of epigenetic factors that mediate interactions between the genes and the environment [6-8].

In this commentary, we will first introduce the basic epigenetic mechanisms and then discuss the results of RA-related epigenetic studies. Finally, we will provide a brief description of epigenetic factor-based future therapeutics in RA.

### Epigenetic regulation

**Figure 1:**
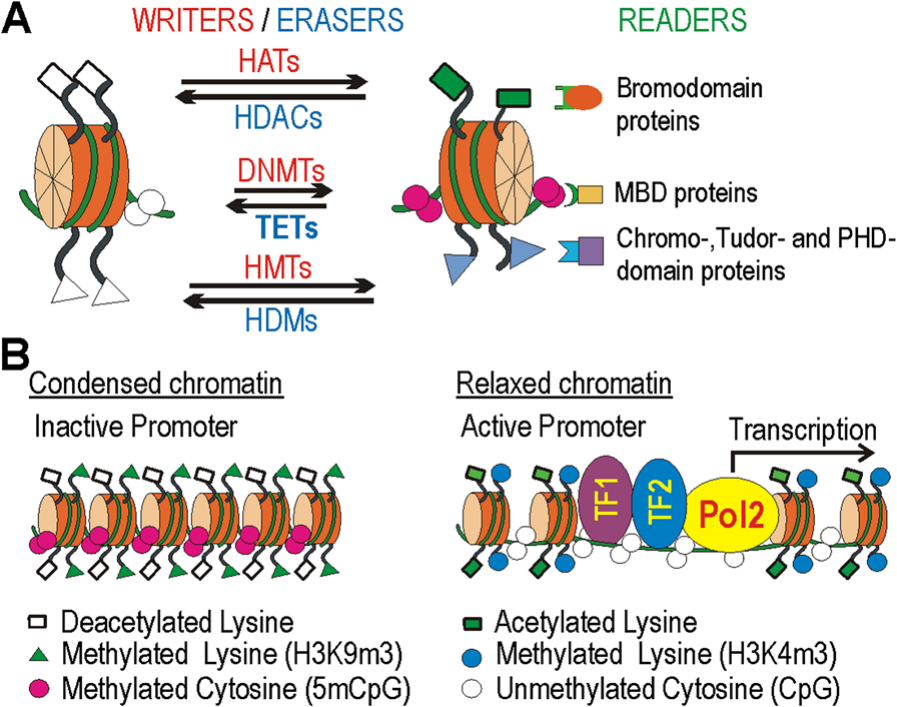
**Schematics of epigenome modifiers and chromatin structure. (A)** Post-translational modifications on histone tails. Epigenetic signal writers are indicated in red, readers in green, and erasers in blue. Acetylated lysine residues are represented by green rectangles, methylated lysines by blue triangles and methylated CpGs of genomic DNA by magenta circles. **(B)** States of the chromatin and associated histone and DNA marks. The figure is original, with some elements adapted from [10]. DNMTs, DNA methyltransferases; HATs, histone acetyltranferases; HDACs, histone deacetylases; MBD, methyl-CpG-binding domain; TET, ten-eleven translocation; TF, transcription factor.

Although there is no ‘carved-in-stone’ definition for epigenetics, it is broadly defined as the study of heritable changes in gene activity that do not involve any alterations in the primary DNA sequence [9]. Epigenetics originally focused on DNA methylation and various histone modifications, but recently expanded to the field of non-coding RNAs. *Ab ovo*, each cell of the body inherits the same genetic information. What makes each cell unique is that, during ontogenesis, different sets of genes are turned on and off. Epigenetic mechanisms establish the proper nuclear milieu for cell-specific gene expression and are responsible for the cellular memory, that is, keeping and transmitting cell-specific gene expression patterns to daughter cells. Epigenetic factors can deposit, interpret and eliminate epigenetic information and, in this sense, they can be divided into distinct functional groups: epigenetic ‘writers’ or enzymes that modify DNA and histones; epigenetic ‘readers’ with specific protein domains that recognize DNA or histone marks; and epigenetic ‘erasers’ that can delete the existing signals to make room for new modifications (Figure 1A).

In studies on cancer and inflammatory and metabolic disorders, frequent errors have been found in epigenetic mechanisms that can result in the miswriting, misreading or faulty removal of epigenetic signals [7].

DNA methylation is catalyzed by DNA methyltransferases (writers) and associated with gene silencing [11]. DNA methylation readers are the methyl-CpG-binding domain proteins, which promote gene silencing by recruiting histone modifiers. Erasers of DNA methylation have been enigmatic for a long time, but recent studies have revealed that demethylation proceeds via selective oxidation of methylated cytosine residues, which is catalyzed by members of the ten-eleven translocation protein family [12,13]. Genomic DNA and associated special nuclear proteins (histones) comprise the nucleosomes that are the building blocks of eukaryotic chromatin and the primary targets of epigenetic modifiers [14]. We briefly describe the two best characterized post-transcriptional modifications because they have been already implicated in RA.

Histone acetylation and methylation exert their effects on gene expression by regulating the accessibility of DNA for transcription factors. As a general rule, modifications decrease the compactness of chromatin structure and promote gene expression (Figure 1B) [14]. Histone acetylation in any position favors transcription activation. Writers are histone acetyltranferases (HATs), erasers are histone deacetylases (HDACs), and bromodomain-containing proteins are the readers of this type of histone modification. Histone methylation represents a diverse set of epigenetic signals [14] for at least three reasons: first, it can occur on various residues (lysine or arginine); second, it exerts its effect on transcription by determining the degree of methylation (that is, mono-, di- or trimethylation); and third, depending on the location of the modified residue, histone methylation can either positively or negatively affect gene expression. Histone methyltransferases, histone demethylases and chromo-, Tudor- or plant homeodomain-containing proteins are the writers, erasers and readers of this type of post-transcriptional modification, respectively (Figure 1A). Different chromatin modifications act together and a highly specific combination of various post-transcriptional modifications creates the histone code that ultimately determines the transcriptional status of a gene [14].

Unlike genomic DNA (that is, genome), epigenetic signals (that is, epigenome) are highly dynamic and show cell type-specific patterns. Each type of cell has its own characteristic epigenome profile with unique gene expression patterns; therefore, studies must be highly specific regarding the investigated cell type.

### Epigenetic alteration in rheumatoid arthritis synovial cells

Early studies found widespread DNA hypomethylation in RA synovial fibroblasts, including hypomethylation of the promoter of the *CXCL12* gene [15] and the LINE1 retrotransposons [16] that are repetitive elements normally repressed by DNA methylation. In these cases, loss of the repressive DNA methylation signal results in increased gene expression. A recent genome-wide study on RA synovial fibroblasts revealed a number of differentially (hypo- and hyper-) methylated genomic regions [17]. Most of the affected genes appear to be involved in inflammation, matrix remodeling, leukocyte recruitment and immune responses [17]. Another study found that the HAT to HDAC activity ratio in arthritic joints was shifted towards HAT dominance, favoring histone acetylation [18], ultimately leading to an increase in gene transcription.

### Epigenetic changes of the adaptive immune system

A genome-wide DNA methylation profiling study in peripheral blood mononuclear cells reported differentially methylated regions in the major histocompatibility complex loci that make a significant contribution to the genetic risk of developing RA [19]. Our group performed the first study on arthritis-related epigenetic modifiers [20], in which chromatin-modifying enzymes were analyzed in B and T cells from arthritic mice and peripheral blood mononuclear cells from patients with RA. All chromatin-modifying enzyme families were represented in the repertoire of genes with arthritis-specific expression, including histone kinases, acetyltransferases, deacetylases, methyltransferases and demethylases, as well as ubiquitin ligases. The most strongly upregulated genes were those encoding Aurora kinase (A and B) enzymes in both arthritic animal and human lymphocytes, and this was accompanied by phosphorylation of serine 10 in the tail of histone H3. This type of histone phosphorylation is a pivotal epigenetic signal for the recruitment of the transcription factor nuclear factor-kappaB (NF-κB) to the promoter of cytokine genes [21], resulting in a cytokine-driven pro-inflammatory response. We found that VX-680, an Aurora kinase-specific inhibitor, significantly reduced the severity of arthritis and promoted B cell apoptosis in the proteoglycan-induced arthritis (PGIA) model of RA. The significance of VX-680-induced B cell apoptosis is that patients with RA who do not respond to anti-tumor necrosis factor therapy are frequently treated with a monoclonal anti-CD20 antibody to eliminate autoantibody-producing B cells [22]. Our findings suggest that drug (VX-680)-induced B cell depletion may provide an alternative to the CD20 antibody-based therapy.

**Figure 2:**
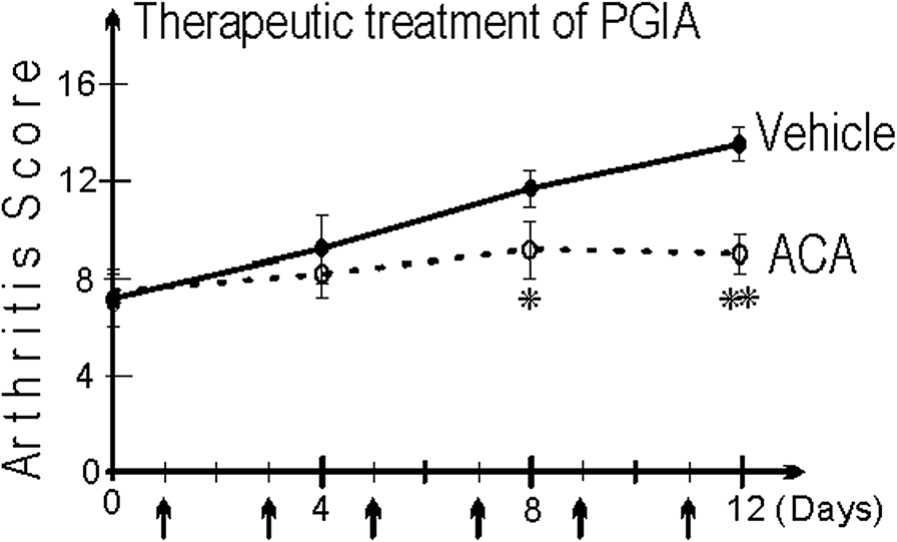
**Therapeutic treatment of established proteoglycan-induced arthritis (unpublished observations).** Arthritic mice (n = 10 per treatment) were divided into two groups with similar mean severity scores and treated with 50 mg/kg anacardic acid or vehicle alone (control) for 12 days. Arrows indicate the days of treatment. The results shown are unpublished observations from original research conducted in our laboratory. Values are the mean ± standard error of the mean. ^*^P <0.04; ^**^P <0.01 ACA-treated versus vehicle-treated groups. ACA, anacardic acid; PGIA, proteoglycan-induced arthritis.

In addition to Aurora kinases, several members of the HAT family are also significantly upregulated in arthritic mice and patients with RA, with the gene encoding Esco2 showing the strongest increase in expression. Esco2 is thought to be required for the establishment of sister chromatid cohesion and it also couples cohesion and DNA replication to ensure that only sister chromatids are paired together [23,24]. Because Esco2 belongs to the HAT family of epigenetic modifiers, it is reasonable to assume that it acts as a selective activator of certain target genes. Anacardic acid (ACA) inhibits HATs [25] and indirectly suppresses NF-κB activation [26]. We tested the therapeutic potential of ACA in mice with established PGIA. Mice treated with ACA displayed significantly reduced arthritis progression as compared to untreated control animals (unpublished observations; Figure 2).

As described earlier, many of the epigenome modifiers can directly or indirectly affect the activity of NF-κB, a master regulator of the transcription of inflammation-related genes. With regard to autoimmune or inflammatory diseases such as RA, the emerging consensus is that epigenetic factors (enzymes) supporting repressive signals are downregulated, whereas those that promote transcription are upregulated. A combination of these activities in immune cells ultimately results in the strengthening of pro-inflammatory pathways and the weakening of anti-inflammatory mechanisms. For example, disease-linked expression of *KDM6B*, a histone methyltransferase responsible for eliminating a repressive epigenetic signal (that is, histone H3 K27 trimethylation), is involved in macrophage activation [27], and repression of the *SETD6* gene, which encodes a known negative regulator of NF-κB, leads to runaway activity of this transcription factor [28].

The results of epigenetic studies in RA raise the question whether the reported epigenetic alterations play a causative role or are the consequences of other pathologic processes that take place in RA. To answer this question, there is a need for further epigenome-wide studies on all types of cells involved in RA, exploration of a larger repertoire of epigenetic signals, and investigation of the epigenetic landscape at different phases of arthritis. It is possible that significant advances will be achieved in the near future because the technologies and model systems, including genome and epigenome-wide analysis tools (such as whole-genome sequencing, chromatin immunoprecipitation sequencing and RNA sequencing) and animal models, are readily available.

Information from RA-associated epigenetic studies can be useful for diagnostic and therapeutic purposes because investigation of the epigenetic landscape can provide both potential biomarkers and therapeutic targets. There have been numerous clinical trials involving patients with cancer that have tested such inhibitors as therapeutics against malignancies [29]. Although we have demonstrated the beneficial effect of specific Aurora kinase and HAT inhibitors [20], and HDAC inhibitors have been tested by other groups [30] in preclinical studies, unlike in the cancer field, there is still no epigenetics-based drug on the market of RA therapeutics.

## Conclusions

A common outcome of genetic and epigenetic mutations is that both ultimately lead to aberrant gene expression. The mechanisms by which genetic mutations affect gene expression are well known, including shorter or longer deletions, insertions, inversions, translocations, or single nucleotide changes within transcription factor binding sites. Mutations hitting genes that encode epigenetic regulators may result in aberrant expression or functional impairment of the affected epigenetic factors [31-33]. The connection between epigenetically provoked and epigenetics-independent genetic mutations is not obvious and is currently under investigation. Both DNA hyper- and hypomethylation can trigger genetic mutations. DNA hypermethylation-mediated silencing of DNA repair genes (for example, *MGMT* and *MLH1*) can result in inactivation of cellular mechanisms responsible for keeping the genetic mutation rate low [34,35] or in induction of microsatellite instability as described in certain types of cancer [36,37]. DNA hypomethylation can reactivate retrotransposons (for example, long and short interspersed nuclear elements), which then promote genetic mutations by inserting extra nucleotides into the exons or regulatory regions of genes [38,39].

Alteration in epigenetic mechanisms can trigger genetic mutations and genetic mutations in epigenetic regulators can lead to an altered epigenetic profile. Therefore, genetics and epigenetics can be considered two sides of the same coin, as has been established in the field of cancer research [40]. It is very likely that in the near future the same conclusion will be reached regarding autoimmune diseases such as RA.

## Abbreviations

ACA: anacardic acid; GWAS: genome-wide association studies; HATs: histone acetyltranferases; HDACs: histone deacetylases; NF-κB: nuclear factor-kappaB; PGIA: proteoglycan-induced arthritis; RA: rheumatoid arthritis.

## Competing interests

The authors declare that they have no competing interests.

## Authors’ contributions

All authors contributed to the manuscript. TAR wrote the first draft and all other authors amended the manuscript. All authors read and approved the final manuscript.

## Authors’ information

TTG and KM are Professors at Rush University Medical Center, and founding members of the Section of the Molecular Medicine. They have been studying immunological aspects of rheumatoid arthritis and ankylosing spondylitis in patients and corresponding animal models for more than three decades. They first described cartilage proteoglycan/aggrecan-induced arthritis (PGIA) and spondylitis (PGISpA) in genetically susceptible mice, and this pioneering work was honored by the Carol Nachman Price. TAR is an Assistant Professor at Rush University Medical Center. He is an expert in disease-associated epigenetic modifications of DNA and histones in cancer, and most recently, in rheumatoid arthritis.
